# Olmesartan is More Effective Than Other Angiotensin Receptor Antagonists in Reducing Proteinuria in Patients With Chronic Kidney Disease Other Than Diabetic Nephropathy^☆^

**DOI:** 10.1016/j.curtheres.2013.02.002

**Published:** 2013-06

**Authors:** Takashi Ono, Toru Sanai, Yoshito Miyahara, Ritsuya Noda

**Affiliations:** 1 Renal Division, Saiseikai Fukuoka General Hospital, Fukuoka, Japan; 2 Renal Division, Tohma Clinic, Koga, Japan; 3 Renal Division, Fukumitsu Hospital, Fukuoka, Japan

**Keywords:** angiotensin II receptor antagonist, nondiabetic chronic kidney disease, olmesartan, proteinuria

## Abstract

**Background:**

Angiotensin II receptor antagonists (ARBs) have a protective effect in patients with chronic kidney disease (CKD) by suppressing progression, possibly by controlling hypertension. One marker of progression in such patients is the degree of proteinuria.

**Objective:**

We aimed to retrospectively examine the protective effect of ARBs (olmesartan, losartan, candesartan, and valsartan) on CKD patients without a history of diabetic nephropathy.

**Methods:**

Data were retrieved from medical records of patients with a diagnosis of CKD (serum creatinine [Cre] <3.0 mg/dL [265.2 μmol/L] and urinary protein of 0.3–3.5 g/g Cre) who were treated with ARBs and those with diabetic nephropathy were excluded. Blood pressure, serum Cre, urinary protein, urinary Cre, and estimated glomerular filtration rate were measured before the research began and at 1, 3, 6, 12, and 24 months after the ARB treatment was started.

**Results:**

Forty-four patients completed the research protocol. Of these, 10 took olmesartan, 13 took losartan, 9 took candesartan, 9 took valsartan, and 3 took telmisartan. Systolic blood pressure was decreased in all cases. The extent of this decrease 1 month after starting ARB treatment was greater for olmesartan than for candesartan (*P* < 0.05), and after 2 years, it was greater than for losartan (*P* < 0.05). Diastolic blood pressure decreased in all patients; this decrease was significantly greater with olmesartan 1 month after treatment started than with candesartan (*P* < 0.05). Olmesartan significantly decreased daily urinary protein compared with that with the other ARBs during follow-up. This decrease 1 month after starting ARB treatment was greater for olmesartan than losartan, valsartan, and candesartan (*P* < 0.01, *P* < 0.01, and *P* < 0.05, respectively), and after 2 years, this effect was still significant (*P* < 0.05, *P* < 0.01, and *P* < 0.01, respectively).

**Conclusions:**

Olmesartan is more effective in reducing urinary protein than other ARBs, suggesting that the renal protective effects of olmesartan may be better than those of other ARBs.

## Introduction

Angiotensin II receptor antagonists (ARBs) confer protection in patients with renal insufficiency, delaying progression in patients with managed hypertension. Most reported cases have been in patients with diabetic mellitus (DM) and related kidney disease.^1^ The Reduction of Endpoints in Non-Insulin-Dependent Diabetes Mellitus With the Angiotensin II Antagonist Losartan trial reported that losartan conferred significant renal benefits in patients with type 2 diabetes and nephropathy.^2^ However, chronic renal failure is not only DM related, but also occurs in other conditions, such as chronic glomerular nephritis and hypertensive nephrosclerosis, and the number of patients with these conditions is equal to the number of patients with DM-related kidney disease.^3^

The current research examined the protective effects of various ARBs in patients with nondiabetic related chronic renal failure.

## Patients and Methods

We used a computerized database of all patients admitted from 2004 to June 2010 for this retrospective review. Patients included in this review were those in whom chronic kidney disease (CKD) had been treated with ARBs and whose diagnosis was made by renal biopsy or their clinical history and results of laboratory investigations. We aimed to exclude patients with nephritic syndrome whose urine protein was >3.5 g/g creatinine (Cre). Internal medicine physicians specializing in diabetes examined patients with diabetic nephropathy earlier than those specializing in the kidney in our hospital. However, they did not examine urine Cre concentrations in those patients. When patients consult internal medicine physicians specializing in the kidney in preparation for dialysis therapy, their CKD is already beyond stage 4 (with a serum Cre concentration >3.0 mg/dL). Because these Cre concentrations were outside the range of our research, those with diabetic nephropathy were excluded. Patients with serum Cre concentrations of 3.0 mg/dL (265.2 μmol/L) and a urine protein of 0.3–3.5 g/g Cre when ARB treatment was started were included in the analysis. To evaluate the effect of each ARB and to ignore the effect of concomitant drugs, when a research patient was taking other drugs (including a calcium antagonist, angiotensin-converting enzyme [ACE] inhibitors [ACEIs], antiplatelet agents, or cholesterol-reducing agents), they were not excluded, provided that they continued to take these drugs after ARB treatment was started and continued to take the same doses during the research period. An arbitrary choice of ARB was not made in any of the patients when they started treatment with an ARB, and each patient took only 1 ARB during the research period. The research was approved by the institutional review board, and informed consent was waived because of the retrospective analysis design.

Blood pressure, serum Cre concentrations, urinary protein concentrations, urinary Cre concentrations, and estimated glomerular filtration rate (eGFR) were measured before the ARBs were started and then at 1, 3, 6, 12, and 24 months and were analyzed retrospectively. All urine and blood Cre concentrations were measured using the creatinase sarcosine oxidase peroxidase method in our hospital. This method for measuring urine and blood Cre concentrations was used throughout this research. The eGFR was calculated using the Japanese Kidney Society method (male eGFR: [mL/min/1.73 m^2^] = 194 × Cre^−1.094^ × age^−0.287^, female eGFR: [ml/min/1.73 m^2^] = 194 × Cre^−1.094^ × age^−0.287^ × 0.739).^4^

Blood pressure, serum concentrations of Cre, urinary protein, urinary creatinine, and eGFR are expressed as the mean (SD). Changes in systolic and diastolic blood pressure and daily urinary protein loss after starting ARB treatment did not follow a normal distribution on a histogram. Because it was assumed that they would closely fit a log-normal distribution, these changes were logarithmically transformed to obtain a normal distribution. The intergroup variation from the start of treatment for hypertension and urine protein concentrations was analyzed using Dunnett’s post hoc test. Comparisons between olmesartan and each ARB (2-group analysis; olmesartan vs valsartan, olmesartan vs losartan, olmesartan vs candesartan, and olmesartan vs other ARBs) for hypertension and urine protein concentrations were analyzed using the Bonferroni adjustment. Multigroup analysis of the 4 groups (olmesartan, valsartan, losartan, and candesartan) for hypertension and urine protein concentrations was performed using repeated-measures ANOVA, assuming a 2-sided 5% significance level and a power of 80%. Statistical analysis was performed using Microsoft Excel 2010 software.

## Results

### Patients’ background

The research protocol was completed by 44 patients with a mean (SD) age of 52.8 (13.4) years; and 20 patients were men. When ARB treatment was started, the mean (SD) systolic blood pressure was 145.3 (22.7) mm Hg and the mean (SD) diastolic pressure was 85.2 (13.4) mm Hg. Mean (SD) serum Cre concentrations were 1.1 (0.6) mg/dL, eGFR was 58.25 (25.2) mL/min/1.73 m^2^, serum potassium concentrations were 4.5 (0.5) mEq/L, and urine protein concentrations were 1.24 (0.86) g/g Cre. The mean (SD) time from the onset of CKD to starting ARB treatment was 9.5 (9.5) years (Table). A renal biopsy was performed in 8 of the 44 patients. Four patients had IgA nephropathy, 2 had membranous nephropathy, 1 had obesity-related nephropathy, and 1 had purpuric nephritis. In the 36 patients in whom a renal biopsy was not performed, the diagnosis was made by the medical history or laboratory test findings. Of these 36 patients, 34 had a diagnosis of chronic glomerulonephritis, and 2 had a diagnosis of hypertensive nephrosclerosis.

Of these patients, 10 took olmesartan, 13 took losartan, 9 took candesartan, 9 took valsartan, and 3 took telmisartan. The mean daily dose at the start of treatment was 13.5 (5.5) mg olmesartan, 28.8 (9.0) mg losartan, 6.7 (1.9) mg candesartan, 48.9 (16.6) mg valsartan, and 33.3 (9.4) mg telmisartan. Because only 3 patients took telmisartan and only 1 completed 2 years of follow-up, those taking telmisartan were excluded from the research. In all patients, the doses remained the same throughout the research period. When ARB treatment was started, there were no significant differences in mean age, systolic and diastolic blood pressure, concentrations of serum Cre, eGFR, serum potassium, and urine protein in any of the groups of patients (Table). The only significant difference was the period between the onset of CKD and the start of treatment with ARB between olmesartan and valsartan (15.8 [9.9] years vs 5.7 [5.4] years, *P* < 0.05) (Table).

### Serum Cre and potassium concentrations and eGFR

In all patients, there were no significant changes in the concentrations of serum Cre and serum potassium and eGFR.

### Systolic and diastolic blood pressure

**Figure 1:**
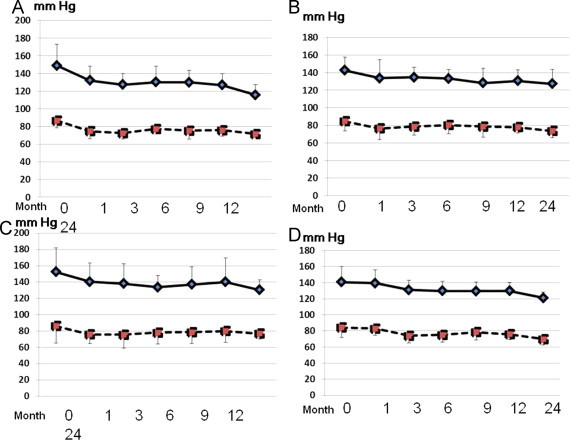
Baseline measurements of systolic and diastolic blood pressure from the start of treatment. Olmesartan (A), losartan (B), valsartan (C), and candesartan (D). Months indicate the time from the start of ARBs. Solid lines, systolic blood pressure; broken lines, diastolic blood pressure.

**Figure 2:**
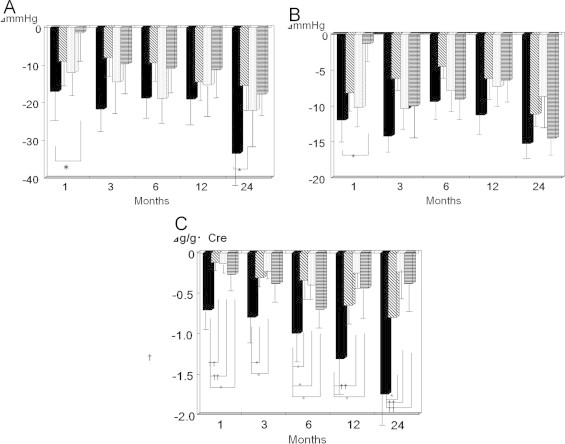
(A) Decrease in systolic blood pressure for patients taking the 4 angiotensin II receptor antagonists (ARBs) that have been logarithmically transformed. Olmesartan and 3 other ARBs were compared. (B) Decrease in diastolic blood pressure for patients taking the 4 ARBs that have been logarithmically transformed. Olmesartan and 3 other ARBs were compared. (C) Decrease in daily urinary protein concentrations that have been logarithmically transformed in patients taking ARBs. Olmesartan and 3 other ARBs were compared. Cre, creatinine. Months indicate the time from the start of ARB treatment. *P < 0.05, ^†^*P* < 0.01. Solid columns, olmesartan; hatched columns, losartan; dotted columns, valsartan; columns with horizontal lines, candesartan.

There were no significant differences in systolic and diastolic pressure by multigroup analysis (ANOVA) among the 4 groups. However, we did observe a time-dependent difference in the decrease in blood pressure between olmesartan and 2 of the ARBs using a 2-group analysis. Systolic blood pressure was decreased in all cases (Figure 1), but the extent of this decrease 1 month after starting ARB treatments was greater with olmesartan than with candesartan (*P* < 0.05) (Figure 2A), and after 2 years, it was greater with olmesartan than with losartan (*P* < 0.05) (Figure 2A).

Diastolic blood pressure decreased in all patients (Figure 1), and the extent of the change was significantly greater with olmesartan than with losartan 1 month after the treatment started (*P* < 0.05) (Figure 2B).

### Daily urinary protein

There were no significant differences in urinary protein by multigroup analysis (ANOVA) among the 4 groups.

**Figure 3:**
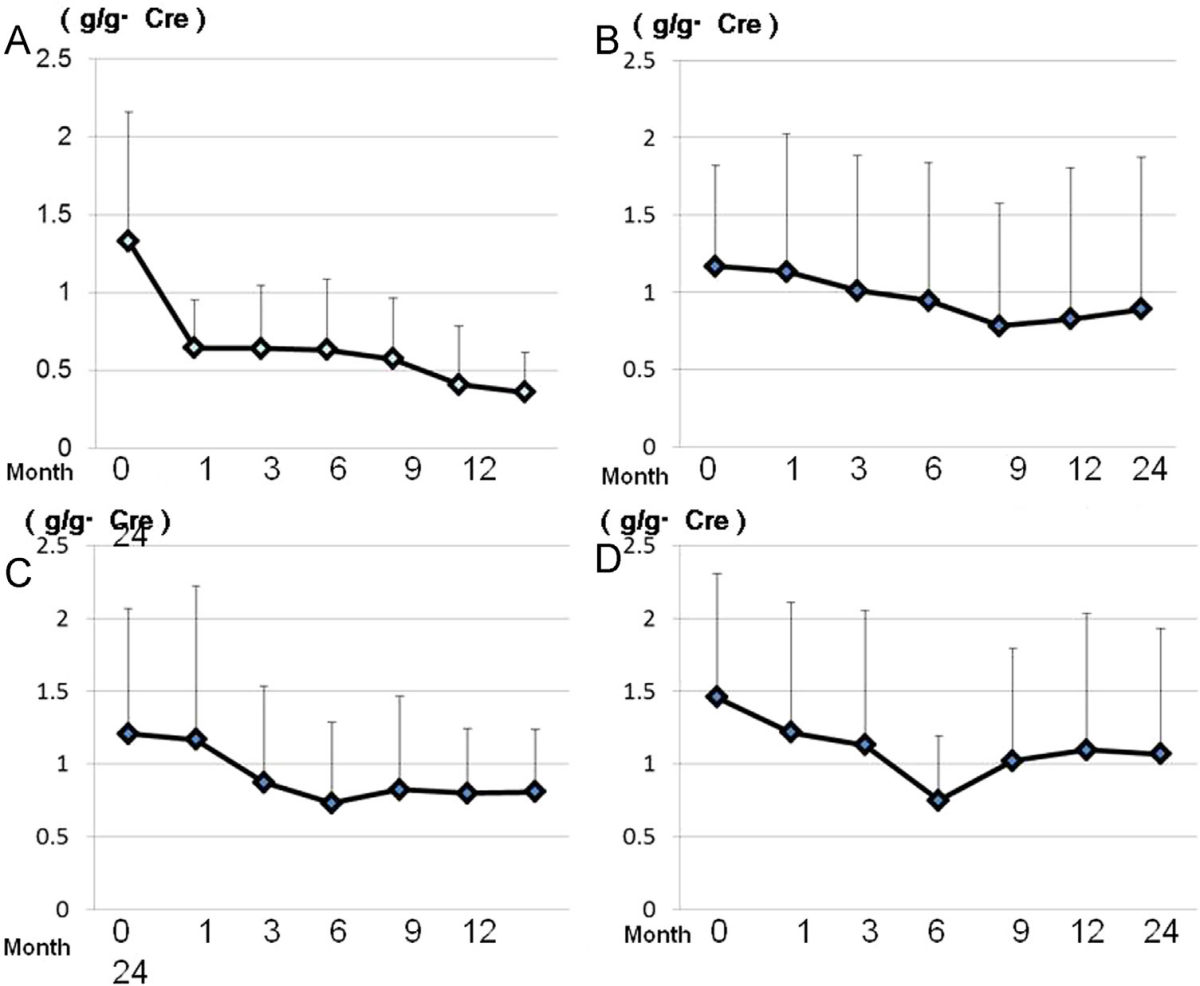
Baseline measurements of urinary protein from the start of treatment. Olmesartan (A), losartan (B), valsartan (C), and candesartan (D). Cre, creatinine. Months indicate the time from the start of ARB treatment.

Urinary protein decreased with ARB treatment in all patients, as shown by using 2-group analysis (Figure 3). Treatment with olmesartan significantly decreased the amount of daily urinary protein loss compared with that with the other ARBs during follow-up. The extent of this decrease 1 month after starting ARB treatment was greater with olmesartan than with losartan, valsartan, and candesartan (*P* < 0.01, *P* < 0.01, and *P* < 0.05, respectively), and after 2 years, this difference was still significant (*P* < 0.05, *P* < 0.01, and *P* < 0.01, respectively) (Figure 2C).

**Figure 4:**
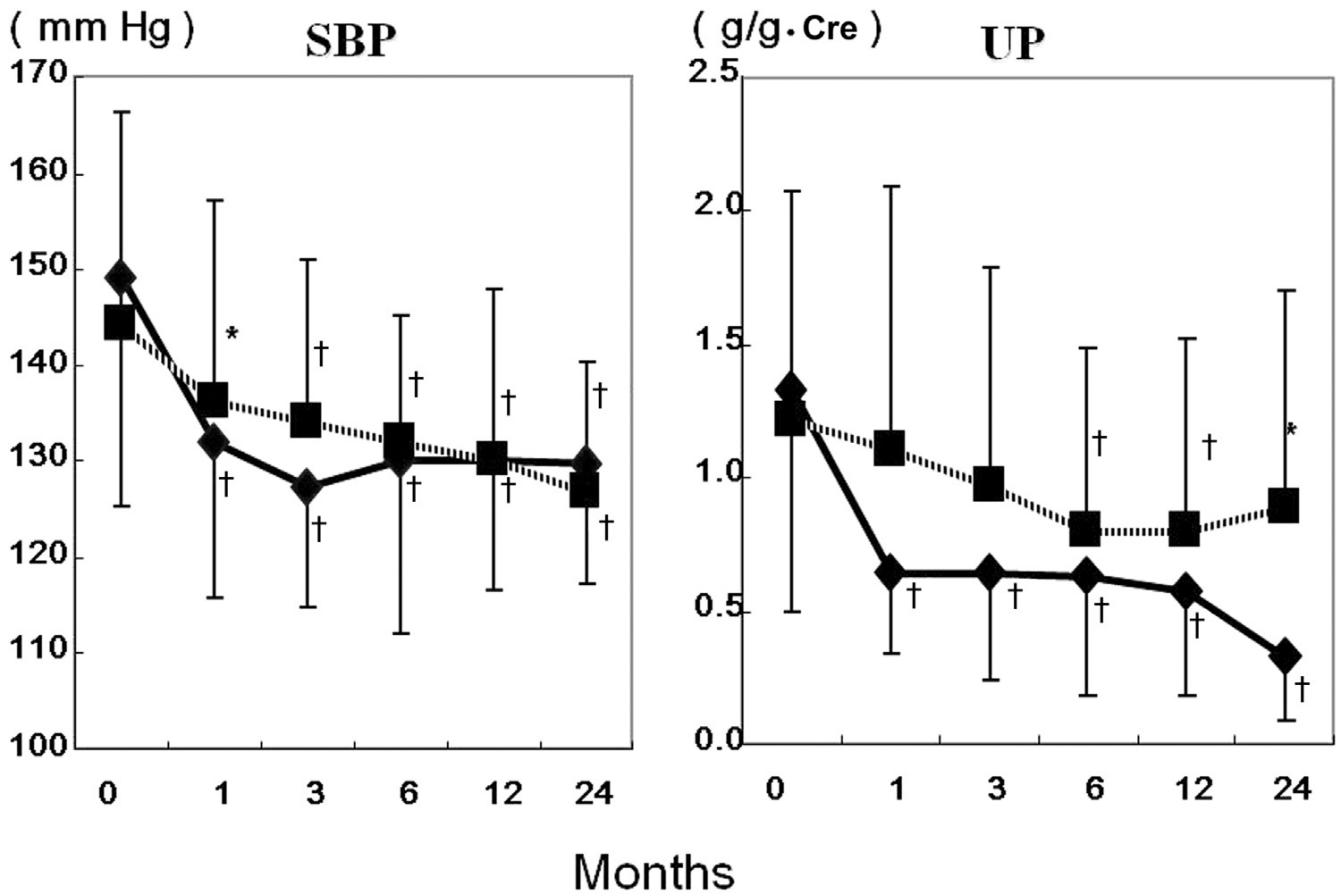
Changes in systolic blood pressure and urinary protein. Olmesartan and 3 other angiotensin II receptor antagonists (ARBs) (valsartan, candesartan, and losartan) were compared. The *P* values were calculated by comparing values after treatment with those when ARB treatment was started. Cre, creatinine; SBP, systolic blood pressure; UP, urinary protein.*P < 0.05, ^†^*P* < 0.01. Months indicate the time from the start of ARB treatment. Solid line, olmesartan; broken line, other ARBs.

Systolic blood pressure and urinary protein significantly decreased with olmesartan as well as with the other ARBs, compared with before starting ARB treatment. The amount of urinary protein was significantly reduced 1, 3, 6, 12, and 24 months after starting oral administration of valsartan, candesartan, and losartan (*P* < 0.01, *P* < 0.05) (Figure 2). Olmesartan significantly reduced the amount of urinary protein at 1 month compared with three other ARBs (valsartan, candesartan, and losartan), and this continued over 24 months compared with that before treatment (*P*<0.01) (Figure 4).

### Daily urinary protein loss in the subpopulation who achieved a blood pressure goal of 130/80 versus those who did not achieve this goal

We evaluated daily urinary protein in the subpopulation who achieved the ARB blood pressure goal of 130/80 at 1 year after starting ARB treatment versus those who did not achieve this blood pressure goal. Urinary protein was decreased in the group who achieved the blood pressure goal compared with the group who did not (olmesartan, achievement group [n = 5]: −0.92 [0.40] g/g Cre vs the nonachievement group [n = 4]: −0.51 [0.30] g/g Cre [*P* < 0.01]; all ARBs, achievement group [n = 27]: −0.56 [0.16] g/g Cre vs the nonachievement group [n = 14]: −0.42 [0.22] g/g Cre [*P* < 0.05]).

## Discussion

According to Japanese Evidence-based Practice Guidelines on CKD in 2009, the number of patients with CKD in Japan exceeded 13 million, and 10 million of these patients had stage 3 disease.^5^ The number of patients on long-term dialysis according to figures from the Society for Dialysis Therapy in 2008 was reported to be 280,000.^3^ The most common causes of end-stage renal disease are diabetic nephropathy (42.9%), chronic glomerulonephritis (25.6%), and nephrosclerosis (9.4%).^3^ According to the CKD clinical practice guidelines, it is recommended that patients with overt proteinuria who have diabetic nephropathy, IgA nephropathy, and nephrosclerosis should be given an ACEI/ARB to maintain their blood pressure at <130/80 mm Hg.^6^ Many of the reports that describe the use of ARBs, which are effective in preserving renal function, relate to patients with diabetic nephropathy.^1,7^ Many studies on nondiabetic chronic renal failure patients have provided evidence regarding the efficacy of ACEIs,^8,9^ but few reports have discussed the use of ARBs.^10–12^

In the current research, we treated nondiabetic patients with various ARBs and evaluated changes in their blood pressure, urinary protein, and renal function. In our patients, blood pressure and urinary protein were reduced by ARB treatment. A reduction in urinary protein suggests a protective effect on kidney function^13^ without causing any side effects. In particular, olmesartan decreased the amount of loss of urinary protein and decreased blood pressure more rapidly than the other ARBs. Even though there were no significant differences in multigroup analysis among the groups (olmesartan, valsartan, losartan, and candesartan), in 2-group analysis (olmesartan vs valsartan, olmesartan vs losartan, olmesartan vs candesartan, and olmesartan vs other ARBs), there was a significant difference within 1 month of starting treatment.

The reason for this difference between olmesartan and the other ARBs is unclear. The reason why urinary protein loss with olmesartan was greater than that with the other ARBs may be because the decrease in blood pressure with olmesartan occurred earlier than that with the other ARBs. There are some reports that olmesartan provides better antihypertensive efficacy than other ARBs.^14,15^ In our analysis, we found that urinary protein decreased more in the achievement group (olmesartan and the other ARBs; blood pressure, 130/80 mm Hg) than that in the nonachievement group. This finding indicates that this antihypertensive effect promotes a decrease in urinary protein. It has also been reported that the antihypertensive action and duration of olmesartan may be greater compared with those with the other ARBs.^16,17^ When comparing olmesartan and the other ARBs, the degree of decrease in urinary protein 2 years after starting ARB treatment was greater than the degree of decrease in blood pressure. Therefore, there may be other reasons why olmesartan reduced urinary protein more than the other ARBs.

Olmesartan has a double-chained domain consisting of carboxyl and hydroxyl groups, which can strongly combine with the ARB type 1 (AT_1_) and block the action of angiotensin II. In addition, the receptor itself depends on inverse agonist activity inhibiting mechanical stress caused by the stretching of cells, which is to be expected in organs such as the heart and kidney.^18,19^

It has been reported that there is an ACE/angiotensin II/AT_1_ receptor axis, as well as an ACE2/angiotensin 1-7/Mas receptor axis, and the balance of both axes leads to a protective effect on organs, such as the heart and kidney.^20^ As a consequence of AT_1_ blockade, ARBs increase angiotensin II levels severalfold above baseline by compensatory feedback.^21^ It has also been shown that olmesartan reduces angiotensin II levels, contrary to expectations, and does not produce aldosterone breakthrough.^22^ Therefore, it was considered that a decrease in angiotensin II does not lead to aldosterone breakthrough.

In Wistar Kyoto rats, olmesartan interacts with ACE2 and increases Ang1-7. Ang 1-7 interacts with the Mas receptor, which has a cardiac and renal protective effect.^23,24^

ARBs, except for olmesartan, have been found to activate ACE2; however, they have to be at much higher doses than olmesartan to activate ACE2.^25^

## Conclusions

Our results suggest that olmesartan decreases blood pressure and protein loss in patients with nondiabetic chronic renal failure more than other ARBs, and this effect may prolong renal function. Additionally, it is possible that the renal protective activity of olmesartan is due to not only an antihypertensive effect but also to other factors. This analysis was retrospective, not a prospective, randomized trial, and the number of patients was too small to confirm the effects of olmesartan. Additionally, a lack of control of concomitant medications was a limitation of this research. Because this was a retrospective evaluation comparing different groups, the medications may have been different and may have enhanced or reduced the response in 1 group versus another. There were no differences among the 4 groups in multigroup analysis (ANOVA). Therefore, another limitation of this analysis is that it was underpowered. The prescription of ARBs was not influenced by the evaluation team. This is also a limitation in that the prescribing physicians could have been biased in their determination of which ARB to use for a given patient.

Therefore, to determine whether olmesartan has a renal protective effect other than an antihypertensive protective effect, a prospective, randomized, large-scale study is necessary.

## Figures and Tables

**Table:** Characteristics of patients who took an ARB and were nondiabetic (N = 41).⁎

	Olmesartan (n = 10)	Losartan (n = 13)	Candesartan (n = 9)	Valsartan (n = 9)	*P*
Sex, M/F	3/7	7/6	5/3	5/4	NS
Age, y	55.3 (11.18)	51.1 (14.2)	50.4 (14.0)	56.4 (13.1)	NS
Dose, mg/day	13.5 (5.5)	28.8 (9.0)	6.7 (1.9)	48.9 (16.6)	—
SBP, mm Hg	149.0 (23.8)	142.6 (14.9)	140.8 (19.3)	152.4 (29.6)	NS
DBP, mm Hg	86.6 (8.1)	84.8 (11.1)	84.2 (12.3)	86.0 (21.1)	NS
Cre, mg/dL	1.1 (0.5)	1.3 (0.4)	1.2 (0.8)	1.1 (0.7)	NS
eGFR, mL/min	54.8 (22.2)	49.1 (15.5)	70.8 (36.4)	61.4 (25.4)	NS
K, mEq/L	4.5 (0.4)	4.3 (0.4)	4.6 (0.5)	4.7 (0.5)	NS
UP, g/g Cre	1.33 (0.8)	1.17 (0.7)	1.46 (0.8)	1.20 (0.9)	NS
Period during ARB treatment being started and diagnosed with CKD, y	15.8 (9.9)	8.1 (5.4)	8.0 (9.4)	5.3 (5.4)†	<0.05†

ARB, angiotensin II receptor antagonist; CKD, chronic kidney disease; Cre, creatinine; DBP, diastolic blood pressure; eGFR, estimated glomerular filtration rate; K, potassium; SBP, systolic blood pressure; UP, urinary protein.

⁎ Values are given as mean (SD).

† Compared with olmesartan.
